# A Systematic Review on the Neuropsychological Assessment of Patients with LBP: The Impact of Chronic Pain on Quality of Life

**DOI:** 10.3390/jcm13206149

**Published:** 2024-10-15

**Authors:** Anna Anselmo, Maria Pagano, Irene Cappadona, Davide Cardile, Fabrizio Russo, Alice Laudisio, Giuseppe Francesco Papalia, Angelo Quartarone, Rocco Salvatore Calabrò, Francesco Corallo

**Affiliations:** 1 IRCCS Centro Neurolesi Bonino-Pulejo, Via Palermo, S.S. 113, C. da Casazza, 98124 Messina, Italy; 2 Operative Research Unit of Orthopaedic and Trauma Surgery, Fondazione Policlinico Universitario Campus Bio-Medico, Via Alvaro del Portillo 200, 00128 Rome, Italy; 3 Research Unit of Orthopaedic and Trauma Surgery, Departmental Faculty of Medicine and Surgery, Università Campus Bio-Medico di Roma, Via Alvaro del Portillo 21, 00128 Rome, Italy

**Keywords:** low back pain, quality of life, assessment, chronic pain, measurement

## Abstract

**Background and objective:** Low back pain (LBP) is most common pain syndrome in Europe, affecting approximately 50% of European citizens. LBP is a complex condition that negatively affects many aspects of an individual’s life such as work productivity, mobility, and mental health. The aim of this study is to assess the impact of pain associated with chronic LBP on patients’ quality of life. **Methods:** Clinical studies reviewed in our search with no time restrictions were identified from PubMed, Web of Science, Scopus, and Cochrane Library databases. Of the initial 1929 studies, only 15 met inclusion criteria. **Results:** Results of our study indicate that chronic pain has a negative impact on numerous functions and areas in which the individual is involved and that this condition leads to reduced performance. **Conclusions:** LBP is a debilitating condition for patients, negatively affecting quality of life. Future studies should focus on validating a standardized assessment that examines all aspects affected by LBP through a customized questionnaire.

## 1. Introduction

Low back pain (LBP) is considered the most common pain syndrome in Europe, affecting about 50% of European citizens. Globally, its annual prevalence has increased from 1.4% to 15.6% over the last two decades [1]. The term LBP refers to a condition of pain, muscle tension, or stiffness localized below the costal margin and above the inferior gluteal folds, with or without sciatica, which is pain radiating from the lower back down the leg [2]. LBP represents a substantial burden for patients and society; in fact, the Global Burden of Disease Study 2019 qualified LBP as one of the leading causes of disability [3].

Almost everyone experiences a brief acute episode of LBP during their lifetime. While many people with back pain recover within a year, some develop a chronic condition with fluctuating or persistent pain of low to medium intensity, interrupted by periods of pain absence or exacerbation. When back pain persists for more than 3 months, it is no longer considered a symptom but a disorder, maintained by factors that may differ from the initial causes. Chronic low back pain (CLBP) can be associated with functional disability and work incapacity, impacting quality of life [4].

LBP has been shown to be multidimensional and associated with negative psychological factors (fear and emotional distress), social factors (stress), lifestyle factors (unhealthy diet and insufficient exercise), and biological factors, including a dysfunctional pain-processing system (central sensitization). In addition to these associations, previous studies have suggested that individuals with LBP have worse performance in problem-solving and working memory and greater difficulty with cognitive tasks than asymptomatic individuals, suggesting an association between LBP and decreased cognitive function [5]. Moreover, in individuals with pain, higher pain intensity has been shown to be associated with lower cognitive performance.

Cognitive impairment is a common comorbidity of chronic pain, significantly compromising patients’ quality of life [6]. Despite this clinically recognized comorbidity, the underlying neuropathological mechanisms remain unclear, although recent preclinical studies have investigated the mechanisms underlying this relationship. Janan Abbas, as well as changes in connectivity and function in brain regions [7]. Patients with chronic back pain report a lower quality of life compared to those without pain, comparable to that of individuals with potentially life-threatening diagnoses.

Back pain is associated with worries and fears, particularly regarding self-identity and social relationships, especially when the pain persists longer than expected [8]. Chronic back pain causes functional disability, anxiety, depression, fearful beliefs about the meaning of pain, work avoidance behavior, stress, increased use of health services, insomnia, the presence of multiple somatic comorbidities and unemployment [9]. 

Given the effect of back pain on quality of life, multidimensional assessment of health-related quality of life is increasingly recognized as essential for the study of back pain [10].

Persistent back pain is considered a health condition influenced by a combination of physical, environmental, cultural, social, and psychological factors. In fact, it is associated with worries and fears, particularly regarding personal identity and social relationships [8]. Furthermore, negative beliefs such as catastrophizing are likely to increase the burden of disease [11]. Therefore, a biopsychosocial approach to chronic LBP is necessary, allowing for a holistic and multidimensional assessment to obtain a comprehensive understanding of the patient’s situation, to best personalize care in a functional and targeted manner [12].

The aim of this study is to evaluate the impact of chronic LBP-associated pain on patients’ quality of life. Examining how pain persistence may impair the ability to perform daily activities, reducing quality of life, and possible correlations with cognitive and psychological alterations, such as deficits in attention, memory, and mood, will contribute to a deeper understanding of the link between chronic pain and general well-being.

## 2. Materials and Methods

### 2.1. Research Strategy

A review of currently published articles in literature was performed and PRISMA guidelines were followed to study cognitive function, psychological dimension, and quality of life in individuals with chronic pain and/or LBP.

The literature search was conducted in several database including PubMed, Web of Science, Cochrane Library, and Scopus, published before May 2024 with no other time restrictions. Key words used were ((((low back pain) AND (quality of life)) AND (assessment)) AND (chronic pain)) AND (measurement). After removing duplicates (n = 713), all articles were evaluated according to title (excluded n = 809), abstract (excluded n = 197), and text (excluded n = 32).

### 2.2. Inclusion Criteria

A study was included if it described or investigated cognitive function, psychological dimension, or quality of life in subjects with LBP or chronic pain associated with this condition. Only studies conducted on human populations and published in English that met the following criteria: (i) original or protocol studies of any type and (ii) articles written in English and published in indexed journals were included in this review.

### 2.3. Exclusion Criteria

A study was excluded if it investigated healthy subjects/patients with clinical conditions other than chronic or LBP or if there was a lack of data about cognitive function, psychological dimension, and or quality of life. Even if their reference lists were checked and included if appropriate, systematic, integrative, or narrative reviews were excluded, as were case reports, dissertations, commentaries, letters, and editorials. No restriction due to year of publication was adopted.

## 3. Results

**Figure 1:**
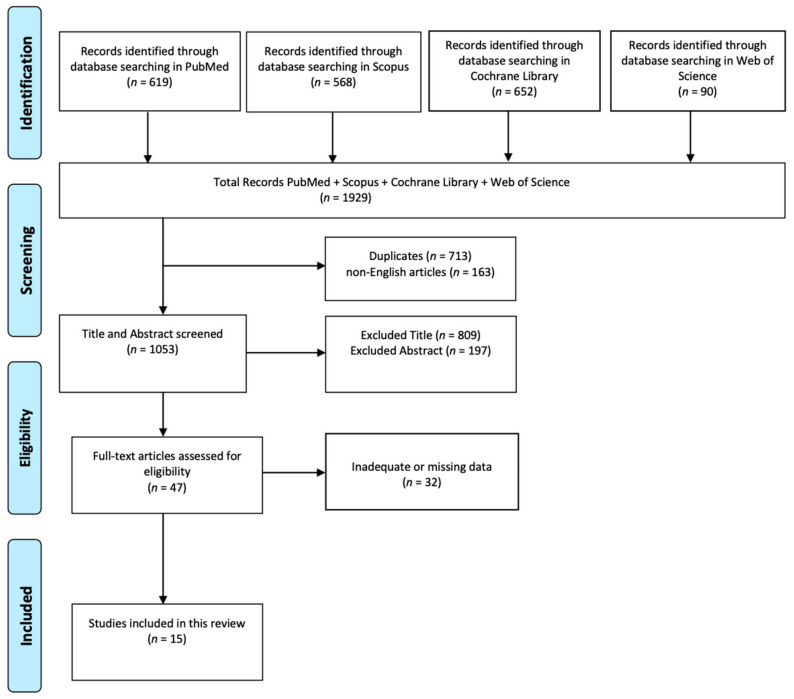
Graph of identification, screening, eligibility, and inclusion of review articles.

The initial electronic search yielded a total of 1929 potentially relevant studies on PubMed, Scopus, Cochrane Library, and Web of Science. After removing all duplicates from the initial list, the articles were evaluated according to title, abstract, and text. The application of these procedures resulted in 15 articles eligible for inclusion among the initial 1929 research articles (Figure 1).

For a detailed description of the 15 included studies, see Table 1.

All 15 selected studies investigated the impact of chronic pain caused by LBP on quality of life. Specifically, five studies [13,14,20,23,26] investigated the possible alteration of cognitive function associated with chronic pain, four [16,18,19,21] investigated all aspects related to quality of life and the degree of disability related to chronic pain, three [22,25,27] examined the psychological dimension concomitant with the pathology, and finally three [15,17,24] examined both the psychological dimension and the sleep quality of subjects with chronic LBP.

### 3.1. LBP and Cognitive Function

Some of the studies considered in our review [13,20,23,26] investigated how chronic pain experienced by LBP sufferers could affect the neuropsychological profile of patients by altering cognitive function, leading to reduced performance.

Some authors [13,26] evaluated the possible alteration of cognitive functions caused by the impact of chronic pain in subjects with chronic LBP. They used the McGill Pain Questionnaire and the Brief Pain Inventory (BPI) to assess the daily pain intensity of patients and compared the scores obtained with neuropsychological evaluations conducted using the Mini-Mental State Examination (MMSE) and the Repeatable Battery for the Assessment of Neuropsychological Status (RBANS) to assess memory, visuospatial abilities, language, and attention. Executive Functions was assessed using the Trail Making Test.

The examination of the relationship between pain severity and neuropsychological performance in subjects with chronic LBP revealed a decrease in attention, visuospatial abilities, mental flexibility, and manual dexterity associated with increased pain severity. Additionally, working memory deficit was found to correlate with the pain intensity experienced by these subjects.

In the studies by [20,23], the authors tried to evaluate whether chronic pain could be associated with specific cognitive deficits that affect daily behavior in subjects with chronic LBP. The neuropsychological profile and executive functions were evaluated using the Cambridge Neuropsychological Test Automated Battery (CANTAB), attention and working memory were investigated through the Wechsler Adult Intelligence Scale (WAIS-IV) and the Stroop Test, and verbal memory and learning were observed with the Hopkins Verbal Learning Test (HVLT) and the Rivermead Behavioral Memory Test (RBMT), and visuospatial abilities were assessed using the Judgment of Line Orientation (JLO) and the Hooper Visual Organization Test (HVOT). Participants demonstrated deficits in measures of attention, working memory, memory, language, and visuospatial performance. This supports and extends previous findings that chronic LBP is associated with cognitive dysfunctions and mild cognitive deficits.

The studies demonstrated that the chronic pain experienced by subjects with LBP could alter various cognitive processes, causing deficits in attention, spatial memory, recognition memory, and decision-making processes.

### 3.2. LBP and Quality of Life

Some authors [16,18,19,21] examined all aspects of quality of life for individuals with LBP, and how chronic pain negatively affects social relationships, work, family relationships, and self-perception. In these studies, the objective was to identify which areas of quality of life were most negatively influenced by chronic LBP.

Through semi-structured interviews and questionnaires such as the SF-36, EQ-5D, WHOQOL, and Roland Morris Disability Questionnaire, all areas and aspects of patients’ daily lives and the level of perceived disability were investigated [16,18,19,21]. The frequency and duration of pain were indeed associated with a reduced quality of life [18].

In the European Union, approximately 44% of individuals aged 55 years and over are affected by pain, which affects their daily activities [28]. This pain has been found to have a severe impact on sleep quality (39.2% of patients), ability to walk (37.4%), routine household chores (33.8%), mood (20.1%), interpersonal relationships (15.3%) and enjoyment of life (16.3%) [29]. The pain was chronic and manifested daily, causing limitations in activities [21]. Participants often reported a negative self-perception in social interactions, with shame and frustration regarding their difficulties in performing daily activities. In some patients, LBP led to a significant loss of social identity, with a perceived inability to perform social roles at home and work.

The most important areas of quality of life affected by chronic LBP for participants were pain intensity, social function, physical function, fatigue, and pain interference [16]. The results revealed a strong association between disability and physical quality of life, indicating that disability negatively and strongly affects the physical quality of life in these patients [19].

These studies confirm how chronic LBP can alter work capacity, the ability to perform normal daily activities, and the maintenance of social and family relationships. The overall quality of life can significantly decrease, leading to further deterioration in physical and mental health.

### 3.3. LBP and Psychological Dimensions

Some authors [22,25,27] have investigated the bidirectional relationship between chronic pain and psychological distress. This means that both factors mutually influence each other, as psychological factors such as anxiety, depression, and stress exacerbate the condition of back pain and chronic pain, which in turn aggravates such clinically significant psychological conditions by fueling catastrophic thoughts that negatively affect quality of life. Using the Symptom Checklist-90 (SCL-90) for psychiatric symptoms, the Beck Depression Inventory (BDI), the State–Trait Anxiety Inventory (STAI), and the Hospital Anxiety and Depression Scale (HADS) for the assessment of depressive and anxious symptoms, pain-related components were investigated through the Fear-Avoidance Beliefs Questionnaire (FABQ), the Health and Pain Questionnaire (HHS), and the Kiel Pain Inventory (KPI), while the Arthritis Self-Efficacy Scale (ASAS) was used to observe avoidance and social withdrawal. The results obtained indicate a connection between the presence of mental distress and almost all aspects of the quality of life in participants with chronic LBP [22,25,27]. In particular, the area of limitation and physical functioning was significantly negatively associated with estimated pain intensity [27]. Patients with LBP experience self-reported symptoms of somatization, anxiety, phobic anxiety, obsessive–compulsive disorder, depression, and hostility [22]. Through the investigations, the authors highlighted how psychological risk factors for chronic LBP, such as depression, anxiety, helplessness, catastrophic thinking, and avoidance behaviors, were positively correlated with pain and disability and negatively correlated with quality of life [25]. Anxiety and depression are not only associated with pain intensity but also partly predict pain intensity in patients with LBP [27]. This constant state of worry can exacerbate the perception of pain, generating catastrophic thoughts according to the fear-avoidance model [30] and con-tributing to a vicious cycle that negatively affects individuals’ quality of life.

### 3.4. LBP and Sleep Quality

In addition to evaluating the psychological dimension of patients with chronic LBP, other authors [15,17,24] focused on the possible deterioration of sleep quality. In all studies, quality of life was assessed using the SF-36, while sleep quality was investigated using the Pittsburgh Sleep Quality Index (PSQI). Additionally, the Work Productivity and Activity Impairment Questionnaire (WPAI) was used to observe the work participation of these subjects [15,17,24]. The results revealed how the chronic pain experienced alters the overall profile of patients causing psychological disturbances that reflect on the general quality of life and sleep dimension [15].

Patients with chronic LBP, in fact, showed significant functional disability and psychological deterioration related to a low quality of life. The results showed that depression among patients with chronic LBP is associated with higher pain scores, lower quality of life, and decreased work productivity related to poor sleep quality and significantly increased anxiety levels [17,24].

The quality of sleep and LBP are linked by a bidirectional relationship. LBP can negatively affect sleep, and in turn, the lack of quality sleep can worsen the lives of patients with LBP. Approximately 25.4% of the participants in the study by Pandelani et al. 2023 [31] reported a severe impact on sleep quality negatively affected by chronic pain, highlighting the challenges they faced in obtaining restorative sleep due to pain-related interruptions that inevitably negatively affect the subject’s work productivity and overall functionality during daily activities.

## 4. Discussion

Chronic LBP is a widely prevalent condition that forces affected individuals to live with chronic pain for most of their lives. The pain experienced has a negative impact on numerous functions and areas in which the individual is involved, such as work, social relationships, leisure activities, family life, self-perception, and their role in all contexts. This condition also affects the quality of sleep and cognitive abilities, such as attention, memory, and processing speed, leading to reduced performance.

Considering the high incidence of the condition and the strong bio-psycho-social impact it has on the patient’s life, with this study we aimed to investigate the topic in depth and comprehensively assess the alterations in function due to chronic pain caused by LBP.

It is crucial to examine not only the physical aspect of pain, but also the repercussions on the patient’s cognitive, psychological, and social functions to better understand the interaction between chronic pain and quality of life and to develop more effective and personalized treatment strategies.

In addition to work, social, and cognitive dimensions, the psychological dimension is also severely affected [32,33]. A vicious cycle of negative and catastrophic thoughts fuels psychological discomforts such as anxiety, stress, and depression, negatively impacting mental and physical health [34,35]. Our results show that there is an association between pain intensity and cognitive function. According to these findings, previous studies [36,37,38,39] that included heterogeneous samples of chronic pain participants reported that individuals with higher pain intensity exhibited greater cognitive impairment compared to those with lower pain intensity in terms of attention, information processing speed, and memory.

It has also been suggested that the association might depend on the complexity of the cognitive task performed, as more complex and demanding tasks compete more for resources. Another explanation could be a slower psychomotor response acting as a weaker moderator between pain and cognitive function [40].

Moreover, in line with our results, numerous cognitive, emotional, behavioral, and environmental factors were involved in the negative perception of chronic pain in patients with LBP [41,42,43,44,45,46,47,48]. Cognitive factors, such as catastrophic pain, expectations of recovery, and pain-related beliefs to avoid fear, have shown an association with the development and maintenance of chronic pain. Psychological factors, such as depression, anxiety, distress, anger, and pain-related fear, are also implicated in this process [49].

Other studies have also highlighted that depression is common in chronic LBP [50,51,52,53] and has been shown to have a prevalence 2 to 3 times higher than the general population. When depression coexists with a chronic medical condition, there is greater functional deterioration, increased symptoms without an identified pathology, higher healthcare utilization, and increased costs [54].

Some previous studies [55,56], in contrast to ours, state that depression is not a response to pain and associated life changes but may also exist prior to the pain. Although it is an interesting point of view, there is currently insufficient evidence to prove that depression is a cause or risk factor for CLBP. Therefore, it is not possible to determine whether depression precedes low back pain or vice versa. Therefore, the results are not entirely consistent across studies, suggesting that depression may predispose someone to the onset of LBP.

CLBP, as we have seen, is a complex condition that negatively affects many aspects of an individual’s life, including physical and cognitive function, psychological and emotional well-being, social integration, coping strategies, work capacity, and sleep [57].

Similarly, sleep disorders are equally complex [58]. Studies by [59,60] have examined how CLBP is associated with sleep problems. Some dimensions of sleep were found to be impaired in patients with CLBP compared to controls (such as sleep duration, sleep disturbances, next-day functionality, sleep quality, and the ability to fall asleep), while others remained unchanged (such as sleep efficiency and sleep activity).

Just as with the inconsistent results in depression, the relationship between LBP and sleep quality is unclear. With more than 50% of people with CLBP reporting sleep difficulties [61], it is unclear whether sleep disturbances are a cause or an effect of chronic pain. Additionally, pre-existing sleep disorders have been correlated with and are strong predictors of higher reported pain intensity [62].

Previous studies have documented that LBP was associated with both physical function and mental well-being [63,64,65]. In fact, the results of these studies showed that chronic pain associated with LBP corresponded with reporting problems in all health-related quality of life domains measured by the EQ-5D-5L [66]. LBP can lead to a decrease in overall perception of health-related quality of life and can impact both physical and emotional functioning [67].

Another important aspect is the somatization of chronicity-related pain [68]. Previous studies suggest that somatization plays a role in LBP outcomes [69,70]. These studies suggest the possibility that somatic symptom burden leads to worse LBP outcomes and lower health-related quality of life (HRQoL). However, these studies do not always consider emotional disorders in patients affected by LBP. Depression is a risk factor for the onset and the chronicization of LBP [71] and somatization often coexists with depression. Therefore, it may be difficult to determine whether the somatic symptom burden predicts LBP outcomes independently of depression.

The results of this study highlight how chronic LBP negatively influences all areas of daily life in which the subject is involved, drastically reducing quality of life. The main strength of our study lies in the fact that, to our knowledge, it is one of the few studies, if not the only one, to comprehensively investigate and unite all the tools used to assess how chronic pain associated with LBP adversely affects all areas and contexts in which the subject is embedded. In particular, our study analyzed the impact of pain on overall quality of life, cognitive function, psychological aspects, and sleep quality assessed by both standardized and ad hoc instruments, which could be further supplemented and improved in the future. This allows the disorder to be reconceptualized from a holistic perspective. This approach could facilitate not only a more personalized and comprehensive treatment for these patients, but also the development of tools or batteries of tests that consider all dimensions associated with chronic low back pain in the evaluation of patients.

However, despite its many merits, this study is not without its limitations. The choice of keywords and/or databases and/or selection criteria may have left some significant articles out of the calculation. The limitation lies in the fact that the sample analyzed is not homogeneous, and the correlation between pain and developed symptoms is not clear from a causal point of view. In addition, there is still no validated standardized assessment to obtain consistent results. Further systematic studies or meta-analyses should address this issue.

## 5. Conclusions

In conclusion, the results of our study highlight how LBP is a debilitating condition for patients, negatively affecting their overall quality of life and affecting every aspect of their lives. This underscores the importance of creating valid and clinically useful tools that comprehensively assess patients with LBP to develop targeted interventions and personalize care.

Living with a chronic disease requires appropriate assessments, preventive measures, and treatments to ensure the best possible quality of life for the patient, helping them to implement the strategies necessary to adaptively cope with all the challenges presented by the disease.

Through this detailed, multifactorial analysis, we hope to contribute to the understanding and improvement of LBP treatment and to stimulate future studies by proposing the development and validation of a standardized assessment tool and a new, purpose-built questionnaire that specifically investigates each area affected by chronic LBP pain that interferes with the subject’s normal functioning and reduces quality of life. This questionnaire should also investigate sexual function, which is poorly assessed but may be affected by this painful condition, to improve the diagnosis and treatment of patients with LBP, with the goal of better quality of life.

Finally, specific studies could be conducted in the future on the impact of integrated quality-of-life treatments with psychotherapy delivered remotely via telemedicine tools and increasingly popular innovative approaches such as wearable devices and virtual reality that can facilitate comprehensive and continuous care of patients, breaking down the logistical barriers associated with traditional care [72].

## Figures and Tables

**Table 1:** Summary of studies included in the research.

References	Aim	Sample	Tools	Results
Weiner et al., 2006 [13]	Assessing cognitive function in subjects with chronic pain caused by chronic LBP	323 patients	MPQ-SF; MMSE; RBANS; TMT; GPT; NART; GDS; SF:36	Examination of the relationship between pain severity and Neuropsychological performance in subjects with CLBP revealed decreases in performance in attention tasks, visuospatial skills, mental flexibility, and manual dexterity that were associated with increased pain severity.
Hnatešen et al., 2022 [14]	Examine the levels of health-related quality of life (HRQoL), pain intensity, and mental distress in participants with chronic LBP	148 patients	SF-36; VAS	The results confirmed the presence of moderate pain and a poorer self-assessment of physical health in comparison to mental health in patients with CLBP
Hong et al., 2014 [15]	To determine health-associated quality of life, sleep disturbances, and psychological status in patients with chronic LBP	47 patients	ODI; BDI; BAI; SF-36; PSQI	Patients with CLBP showed considerable functional disability and significant impairment of psychological status with a low quality of life
Eilayyan et al., 2023 [16]	Identify which domains of HRQoL are most important from the perspective of individuals with chronic LBP.	26 subjects	PROMIS	The most important domains of HRQoL perceived by participants were pain intensity, social function, physical function, fatigue, and pain interference.
Tsuji et al., 2016 [17]	Investigate the impact of depression on HRQoL in CLBP and to assess the relationship between depression and work impairment and healthcare use among CLBP patients	30.000 subjects	NRS; SF-36; WPAI	Depression among CLBP patients was associated with higher pain scores and lower HRQoL scores, as well as lower labor productivity and increased healthcare use.
Ludwig et al., 2018 [18]	Investigating the effects of LBP, type of symptoms, activity limitations, frequency, duration, and severity on health-related quality of life.	707 subjects	EQ-5D	The frequency and duration of pain were associated with reduced quality of life
Bailly et al., 2015 [19]	Better understand experiences of patients living with chronic LBP, with a focus on impact on relationships with family, friends, and work colleagues.	25 subjects	Non-standardized ad hoc test on QoL	Participants often reported negative self-perception in social interactions, with shame and frustration at their difficulties in performing activities of daily living.
Schiltenwolf et al., 2017 [20]	Assess whether chronic pain may be associated with specific cognitive deficits that affect daily behavior.	33 subjects	MWT-B; WAIS III; TMT; PRM; SSP; CANTAB; CRT; HADS	Cognitive changes found in patients with LBP. Chronic pain appears to impair both information processing and working memory
Stefane et al., 2013 [21]	Assessing perceived pain, disability, and quality of life in individuals with chronic LBP	97 subjects	RMDQ; WHOQOL-Bref	The intensity of perceived pain was considered high, the level of disability experienced was considered severe, and the area of physical quality of life appeared to be the most impaired and strongly associated with the level of disability.
Christensen et al., 2015 [22]	Assessing mental distress among patients with LBP	770 patients	SCL-90; SF-36; RMDQ; FABQ	Patients with LBP experienced self-reported symptoms of somatization, anxiety, phobic anxiety, obsessive–compulsive behavior, depression, and hostility.
Corti et al., 2021 [23]	Exploring the cognitive profile of people with chronic LBP	31 subjects	CANTAB; WAIS-IV; HVLT; BNT; JLO; HVOT; SF-MPQ; VAS; RMDQ; DASS-21; SF-36	Participants with CLBP had negative scores on measures of attention/working memory, memory, language, and visuospatial performance.
Depreli et al., 2021 [24]	Assessing anxiety, quality of life, and sleep quality in patients with chronic neuropathic LBP.	83 subjects	SF-36; STAI; PSQI	Neuropathic LBP was associated with poor quality of life, poor sleep quality, and significantly increased anxiety level
Scholich et al., 2012 [25]	Examine correlations between the outcome variables of pain intensity, disability, and HRQoL and between these outcomes and known psychological risk factors for chronic LBP.	52 subjects	CPG; BDI; STAI; KPI; TSS; HHS; CTS; ASAS; BES	Psychological risk factors for CLBP were positively related to pain and disability and negatively related to HRQoL.
Simon et al., 2016 [26]	To determine how working memory and pain catastrophizing are associated with measures of daily pain intensity in subjects with chronic LBP	60 subjects	BPI; BPS; WAIS-IV; PCS; MMSE	Working memory was impaired in correlation with the intensity of pain experienced by individuals with chronic LBP in daily life
Mok and Lee 2008 [27]	Examine the relationship between anxiety, depression, and pain intensity in patients with LBP	102 subjects	HADS; NRS	Anxiety and depression were not only associated with pain intensity, but they also, partly, predicted pain intensity in patients with LBP

Legend: SF-MPQ: McGill Pain Questionnaire short form; MMSE: Mini Mental State Examination; RBANS: Repeatable Battery for the Assessment of Neuropsychological Status; TMT: Trail Making Test; GPT: Grooved Pegboard Test; NART: National Adult Reading Test; GDS: Geriatric Depression Scale; SF-36: Short Form Survey; VAS: Visual Analogue Scale; ODI: Owestry Disability Index; BDI: Beck’s Depression Inventory; PSQI: Pittsburgh Sleep Quality Index; PROMIS: Patient-reported Outcomes Measurement Information System; NPRS: Numeric Pain Rating Scale; WPAI: Work Productivity and Activity Impairment; PROMs: Patient-Reported Outcome Measures; SCL-90-R: Symptom Check List Revised; MPI: Multidimensional Pain Inventory; EQ-5D: EuroQol-5D; MWT-B: Multiple Choice Word Test-B; WAIS III: Wechsler Adult Intelligence Scale III; PRM: Pattern Recognition Memory; CANTAB: Cambridge Neuropsychological Test Automated Battery; CRT: Cognitive Reflection Test; HADS: Hospital Anxiety and Depression Scale; RMDQ: Roland Morris Disability Questionnaire; WHOQOL-Bref: World Health Organization Quality of Life-Bref; FABQ: Fear-Avoidance Beliefs Questionnaire; WAIS IV: Wechsler Adult Intelligence Scale IV; HVLT: Hopkins Verbal Learning Test; BNT: Boston Naming Test; JLO: Judgment of Line Orientation; HVOT: Hooper Visual Organization Test; SF-MPQ: Short-Form McGill Pain Questionnaire; DASS-21: Depression Anxiety Stress Scale Short Version; STAI: State–Trait Anxiety Inventory; CPG: Chronic Pain Grade Questionnaire; KPI: Kiel Pain Inventory; CTS: Catastrophizing Thoughts Scale; ASAS: Avoidance of Social Activities Scale; HHS: Help–Hopelessness Scale; TSS: Thought Suppression Scale; BES: Behavioral Endurance Scale; BPI: Brief Pain Inventory; BPS: Back Performance Scale; PCS: Pain Catastrophizing Scale; NRS: Numerical Rating Scale.

## Data Availability

Datasets are available to download on request. Requests should be directed to the corresponding author.
